# Human Brain Basis of Musical Rhythm Perception: Common and Distinct Neural Substrates for Meter, Tempo, and Pattern

**DOI:** 10.3390/brainsci4020428

**Published:** 2014-06-17

**Authors:** Michael H. Thaut, Pietro Davide Trimarchi, Lawrence M. Parsons

**Affiliations:** 1 Center for Biomedical Research in Music, Colorado State University, Ft. Collins, CO 80523, USA; 2 Department of Psychology, University of Sheffield, Sheffield S102TP, UK; 3 Department of Psychology, University of Milano-Bicocca, Milan 20126, Italy

**Keywords:** music, neuroimaging, rhythm, perception, brain

## Abstract

Rhythm as the time structure of music is composed of distinct temporal components such as pattern, meter, and tempo. Each feature requires different computational processes: meter involves representing repeating cycles of strong and weak beats; pattern involves representing intervals at each local time point which vary in length across segments and are linked hierarchically; and tempo requires representing frequency rates of underlying pulse structures. We explored whether distinct rhythmic elements engage different neural mechanisms by recording brain activity of adult musicians and non-musicians with positron emission tomography (PET) as they made covert same-different discriminations of (a) pairs of rhythmic, monotonic tone sequences representing changes in pattern, tempo, and meter, and (b) pairs of isochronous melodies. Common to pattern, meter, and tempo tasks were focal activities in right, or bilateral, areas of frontal, cingulate, parietal, prefrontal, temporal, and cerebellar cortices. Meter processing alone activated areas in right prefrontal and inferior frontal cortex associated with more cognitive and abstract representations. Pattern processing alone recruited right cortical areas involved in different kinds of auditory processing. Tempo processing alone engaged mechanisms subserving somatosensory and premotor information (e.g., posterior insula, postcentral gyrus). Melody produced activity different from the rhythm conditions (e.g., right anterior insula and various cerebellar areas). These exploratory findings suggest the outlines of some distinct neural components underlying the components of rhythmic structure.

## 1. Introduction

The perception and performance of music requires the ability to build a temporally ordered architecture of sound sequences in rapid succession. The complex processes underlying this ability have attracted accelerating research in ethology, developmental cognitive sciences, experimental psychology, neuroimaging, and behavioural neurology [1,2,3,4]. Musical experiences involve complicated interactions amongst a variety of cognitive, perceptual, affective, and motor processes. Recent neurological and neuroimaging data suggest that distinct neural systems subserve the melodic, harmonic, timbral, affective, and rhythmic aspects of music [5,6,7,8,9,10,11,12,13]. However, a closer look at the structure of these musical elements reveals that the single element of rhythm is also not considered a singular unified component but a composite of temporal sub-elements which all contribute to the organization and perception of rhythm in music [14,15,16,17]. Therefore, the question arises whether it is the case that not only are separate musical elements subserved by distinct neural systems but also within a single musical element such as rhythm, distinct neural systems underlie the separate aspects of time processing within musical rhythm perception.

Rhythm is music’s central organizing structure. It orders the movement of musical patterns in time. Rhythm is indispensable for music. Whereas rhythm can exist without melody or harmony, melody and harmony cannot exist without rhythm. However, definitions of “rhythm” have frequently been identified with one of its sub-constituent elements such as meter, beat, or tempo. Most definitions in musicology, however, consider musical rhythm—at least within Western music—a hierarchically distributed composite of temporally organizing elements, consisting of four fundamental elements: (1) the basic unit of time or tactus (repetition of identical short duration periods marked on/off by beats); (2) the frequency of the tactus (tempo); (3) cyclical groupings of beats into units marked by accents (meter); and (4) rhythmic patterns or gestures (sequences of time intervals that may or may not extend across meter units). We relied on the foregoing constituent components of rhythm in designing the stimuli for our study of functional brain activity during the perception of sub-components of musical rhythm [18]. There has been a broad line of insightful studies of different aspects of rhythm perception, as well as of the contrasts between rhythm perception and other musical elements. However, definitions of rhythm—and consequently the design of rhythmic stimuli—have not always been consistent across studies, making the comparisons between experimental rhythm conditions difficult. Our study is attempting to contribute to this line of inquiry by studying the neural basis of musical rhythm perception by contrasting three musicologically-defined components of hierarchic rhythm—beat patterns, meter patterns, tempo patterns—and using rhythmic stimuli based on standardised music perception tests (see Experimental Section).

Although previous investigations suggest that rhythm is not processed in a single area but in distributed brain areas, a systematic comparison of the foregoing components of rhythm has not been undertaken. Evidence for distributed networks of brain areas being involved in rhythm perception comes from several different lines of investigation. For example, comparing rhythmic beat patterns to other elements of music, neuroimaging and neurological data imply that the neural systems subserving the perception and production of rhythmic beat patterns are different from those underlying pitch, melody, timbre, and tonality [5]. Functional brain activation studies have also examined perceptual discrimination and passive listening tasks for rhythmic sequences, as compared to sequences of other musical features such as melody, timbre, or pitch. A PET study of non-musicians performing perceptual same-different discrimination of pairs of rhythmic patterns of 5–10 notes and 2.5 s in duration, isolated activity in left insula and Broca’s area, when compared to timbre and pitch data [19]. In an fMRI study of non-musicians passively listening to musical rhythms, activations were observed in bilateral planum temporale, left SMA, bilateral pre-motor cortex, and bilateral lobule VI in cerebellum [20]. In addition, an fMRI study of non-musicians passively listening to isochronous metric and non-metric drum patterns observed activity in dorsal premotor, SMA, pre-SMA, and lateral cerebellum for predictable sequences, with more complex patterns recruiting increased activity in superior prefrontal cortex [21]. With respect to tempo, two recent fMRI studies of non-musicians listening to drumming patterns provide evidence that (a) individual preferences for specific tempos are associated with increased activity in ventral pre-motor cortical areas and (b) greater activation in a range of cortical areas (left BA 44, insula, BA 47, SMA, and BA 6) during rhythm production is associated with individuals who more readily engage with rhythm [22,23]. In addition, an fMRI study comparing musicians and non-musicians passively listening to simple drum patterns (*versus* a random rhythm) reported a common network of activations in bilateral superior temporal cortex, left inferior parietal cortex, and right frontal operculum, as well as greater activity in musicians in left perisylvian areas as compared to non-musicians [24]. Similar findings were observed in a more recent fMRI study of a group of musicians and non-musicians attending to complex metric rhythms [25].

A related line of neuroimaging research has focused on the perception of the duration of auditory stimuli. Judgments of interval duration—*i.e.*, time elapsing between beats—are critical for rhythm perception as they create the basis for the anticipatory time structure for periodic beat sequences. For example, an fMRI study of non-musicians discriminating sounds of different duration implicated right inferior parietal cortex, bilateral premotor cortex, and dorsolateral prefrontal circuits [26]. These observations were interpreted respectively to subserve time-dependent attention, working memory functions necessary for perception of duration of auditory stimuli, and comparison of time intervals. Other neuroimaging studies of this kind suggest a role for basal ganglia, and possibly prefrontal, pre-motor, and cerebellar areas, in explicit timing processes, as well as inferior parietal and pre-motor cortical areas in expectation-based processes [27,28,29,30,31,32]. Furthermore, an MEG study [33] found that the amplitude of M100 auditory field potentials located by dipole analysis in Heschl’s Gyrus changed in direct correspondence with changes in durations of rhythmic time intervals: increases in durations showed increases in M100 amplitudes and decreases showed the reverse. These neural responses were present for duration changes below and above the level of conscious perception, implicating a role for primary auditory cortex for rhythmic interval processing in the millisecond range.

Complementing the neuroimaging studies are various neuropsychological investigations of meter, rhythm pattern, and time perception. One line of research has examined the dissociation of rhythm processing from the processing of melody. These studies report meter and rhythmic pattern processing can be spared in patients with impaired melody processing (left or right temporal lesions; tone deaf individuals) [34,35]. Another line of studies has examined the processing of meter as compared to rhythmic pattern. One model of meter representation assumes a hierarchical organization whereby meter (a more global feature) is induced on the basis of pattern (a more local feature) [36]. Findings with neuropsychological patients have been generally more consistent with a model of independent (non-hierarchical) processing whereby patients’ meter processing is spared in spite of impaired rhythmic pattern processing or their rhythmic pattern processing is spared in the face of impaired meter processing [37,38,39]. Related differences are observed in ERP data for perception of meter and rhythm pattern by both musicians and non-musicians [40]. Left inferior parietal lobule is implicated for processing rhythm pattern, whereas meter processing is affected by damage to either anterior superior temporal or basal ganglia and tempo processing is affected by damage to basal ganglia [38,39,41,42,43]. Neuropsychological studies of time duration and perception implicate circuitry in right hemisphere areas of inferior parietal cortex, pre-motor cortex, and dorsolateral prefrontal cortex, implementing time-dependent attention and working memory functions that would be necessary for perception of duration of auditory stimuli [44,45]. Other lines of neuropsychological research have suggested a possible role for the cerebellum in the perception of auditory duration, but the evidence is somewhat inconsistent. One set of patient studies supports this hypothesis but others do not [46,47,48].

In summary then, various investigations of the neural basis of musical rhythm perception and production implicate distributed networks of brain areas. The aim of the present study is to clarify which areas specifically are responsive to particular rhythmic components of music, *i.e.*, pattern, meter, and tempo. The processing of rhythmic sub-components requires distinct computational processes. For example, processing rhythmic patterns (or phrases) involves representing temporal intervals at each local time point which vary across segments and must be linked at a higher level of temporal organization and sequencing, whereas processing meter involves representing repeating cycles of strong and weak beats, and processing tempo requires representing the change in the rate of sounds. By contrast, perceiving melody, which is also examined in the present study, involves processing pitch interval, pitch height, melodic contour, tonal centre, phrase structure, and harmonic structure. These distinct computational requirements likely engage different neural mechanisms, consistent with relatively sparse comparisons in prior studies. The present study attempts to provide a more comprehensive and comparative data set for assessing these predictions, particularly on a within-subject basis (rather than across different studies and labs). Furthermore, comparative musicology has long recognized the great diversity of rhythmic systems across musical cultures [49,50]. Metrical organization in meter systems, as it has commonly emerged in Western music during the Renaissance, is not present, for instance, in West African polyrhythmic music or in Indian Raga music. The neural underpinnings for the ability to use relatively independent modular approaches across different cultures to build idiosyncratic rhythmic architectures are not known. It was hoped that the current investigation could also shed light on this question.

We used PET to measure functional brain activity in individuals making covert same-different discriminations of pairs of rhythmic auditory patterns. One advantage of the PET environment is that there is no acoustic noise of any kind during task performance and during the acquisition of localized brain blood flow measures, as compared to typical fMRI settings (which is typically loud and rhythmic). We examined the selective perception of pattern (phrasing), tempo (dynamically increasing or decreasing rate), and meter (e.g., the differing periodicities of 3/4, 4/4, 5/4, 5/8, 7/8, 9/8). We included a melody discrimination task in which subjects compared pairs of isochronous auditory sequences in which it was possible for a single note to vary in pitch in one of the two melodies. Thus, by design, the temporal task stimuli have constant pitch but variability in meter, phrasing, and tempo, while the melody task stimuli have constant meter, phrasing, and tempo but variability in pitch (melody). In addition, we evaluated these tasks across a range of musical skill by including expert musicians and non-musicians. The tasks and stimulus materials were adjusted in musical rhythmic complexity and subtlety so that musicians and non-musicians performed at comparable levels of accuracy (as in other studies, e.g., [51]). The emphasis was on natural musical features found universally in human culture, rather than isochronous or non-musical rhythmic sequences.

## 2. Experimental Section

### 2.1. Subjects

Participating in the study were five musicians with (at least) an undergraduate university degree in music, and five non-musicians with no music training or performance experience beyond childhood. Each individual was right-handed, as confirmed by the Edinburgh Handedness Inventory (Oldfield, 1971). All ten subjects were healthy with no history of psychiatric or neurological disorders. Non-musician (mean age of 23 year, 19–27 year) and musician (mean 35 year, 26–44 year). Subjects gave written, informed consent.

### 2.2. Stimuli and Task

**Figure 1:**
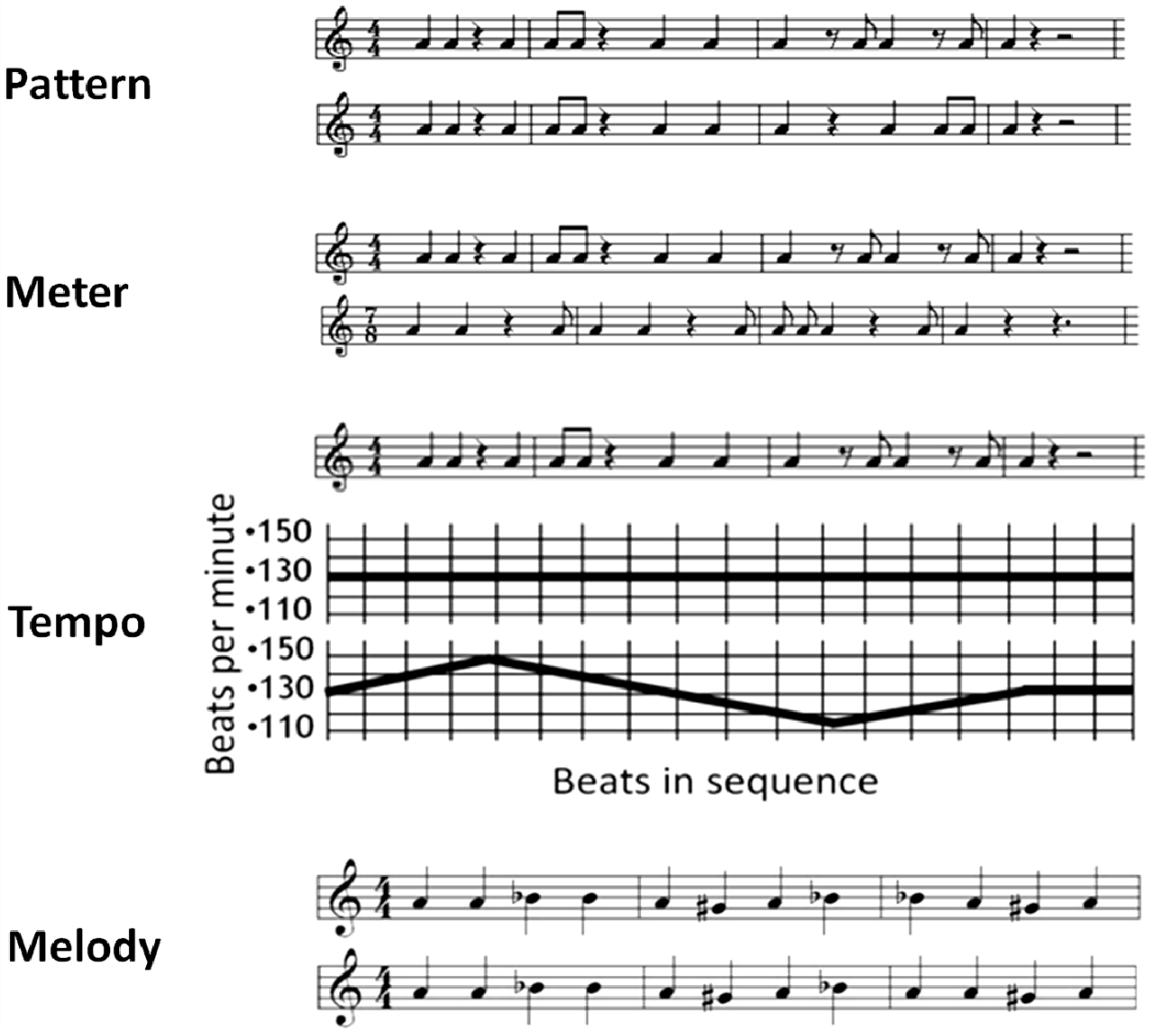
From top to bottom: a sample of a pair of stimuli for a trial in the Pattern task in which there are different patterns; a sample of a pair of stimuli for a trial in the Meter task in which there are different meters; a sample of a pair of stimuli for a trial in the Tempo task in which there are different tempos; and a sample of a pair of stimuli for a trial in the Melody task.

The experimental tasks included conditions in which participants discriminated the rhythmic elements of pattern, meter (e.g., the differing periodicities of 3/4, 4/4, 5/4, 5/8, 7/8, 9/8), and tempo (dynamically increasing or decreasing rate) (see Figure 1). In the meter task accents on the first beat of a meter unit assured perception of the complex meter as a single metric unit and not as a sequentially alternating compound of 2 less complex meters (e.g., 4/4 followed by 3/4). The stimuli were always 440 Hertz computer-generated piano timbre sounds of 231 or 462 ms duration. The interval between tones in a stimulus sequence was a multiple of 231 ms. Stimuli on the pattern trials were modelled on the standardized Seashore Test (1938); the stimuli on the tempo and meter trials were modelled on the Gordon Musical Aptitude Profile (1995) [52,53]. These stimulus materials were adjusted (*i.e.*, in tempo or accentuation) so that the stimuli on the pattern, meter, and tempo trials were as similar as possible. In the melody task, the tone alternated between 415, 440, and 466 Hertz in sequences of twelve 462 ms (quarter note) tones, without rests (*i.e.*, all notes had the same duration) (see Figure 1). When the melodies were different on a trial, a single note varied in pitch in one member of the stimulus pair. The melody stimuli required a comparable degree of cognitive demand with respect to auditory perception, working memory, and comparison and decision processes as did the rhythm tasks, without requiring the processing of varying rhythmic features. The stimuli in the rhythm tasks had constant pitch but variability in meter, phrasing, and tempo; the melody task stimuli had constant meter, phrasing, and tempo, but variability in pitch (melody). Pattern, meter, tempo, and melody stimuli had between 9 and 12 events. A “different” trial contained the same number of events (and accents) and was of the same total duration as the “same” trials.

Participants made covert discriminations of pairs of rhythmic auditory patterns. Before the start of each scanned set of trials, volunteers were informed of which kind of task was being performed: *i.e.*, on which feature of the stimuli he/she should focus their attention (one of three principal components of rhythm, or melody). Different conditions varied with respect to the exact nature of the task being performed, with stimuli being in almost all respects very similar across conditions. Subjects were instructed to compare the first and second sequence of sounds on each trial to discriminate whether they were same or different with respect to the feature highlighted in the task condition. In all other respects, the instructions for each task were identical. Volunteers were instructed to make a covert response of same or different at end of each trial. Across all conditions, the inter-sequence interval was 1000 ms and the inter-trial interval was 1750 ms; the trial time was 14 s on average. Stimuli were adjusted in structural complexity to produce comparable mean accuracy on experimental pre-testing prior to the study of musicians and of non-musicians on each task at 70% correct rate. With the two groups performing at a comparable level of accuracy and effort, we assumed that there would be less risk of a confound due to a difference in effort or task difficulty. Such a difference might skew the functional brain activity in ways difficult to interpret, complicating comparisons between group mean data. In the post-scan testing, we confirmed that the two groups’ mean accuracy for each condition was comparable: musicians’ mean accuracy (and standard deviation) for pattern, meter, tempo, and melody respectively was 0.82 (0.84), 0.84 (1.14), 0.86 (0.55), 0.84 (1.14); non-musicians’ accuracy was 0.78 (1.3), 0.78 (0.84), 0.82 (0.84), 0.78 (0.84).

### 2.3. Procedure

Each subject subsequently performed nine PET trials: two trials each of the pattern task, meter task, tempo task, and melody task, as well as one rest trial. The subject’s eyes were closed on all trials. During each 60 s of task there were four covert same-different discrimination judgments. For half of the trials, the pairs of stimuli were identical. After the PET session, subjects replicated the trials from the PET session overtly indicating their responses.

### 2.4. Image Acquisition

PET scans were performed on a GE 4096 camera which has a pixel spacing of 2.0 mm, an inter-plane, centre-to-centre distance of 6.5 mm, 15 scan planes, and a z-axis field of view of 10 cm. Correction for radiation attenuation was made by means of a transmission scan collected before the first scan using a 68Ge/68Ga pin source. Cerebral blood flow was measured with H215O (half-life = 123 s), administered as an intravenous bolus of 8–10 mL of saline containing 60 mCi. At the start of a scanning session, an intravenous cannula was inserted into the subject’s left forearm for injection of each tracer bolus. A 30-s scan was triggered as the radioactive tracer was detected in the field of view (the brain) by increases in the coincidence-counting rate. During this scan, the subject performed a task in one of the three conditions. Immediately following this, a 60 s scan was acquired as the subject lay with his/her eyes closed without performing a task. The latter rest PET images, in which task-related rCBF changes are still occurring in specific brain areas, are combined with the task PET images in order to enhance detection of relevant activations. Following the latter scan, subjects performed one final (fifth) trial (without being scanned). A 10-min inter-scan interval was sufficient for isotope decay (5 half-lives) and return to resting state levels of regional blood flow within activated regions. PET data were reconstructed using a Hann filter, resulting in images with a spatial resolution of approximately 7 mm (full-width at half-maximum (FWHM)).

Anatomical MRI scans were performed on an Elscint 1.9 T Prestige system. The scans employed 3D Gradient Recalled Acquisitions in the Steady State (3D GRASS), with a repetition time of 33 ms, an echo time of 12 ms, and a flip angle of 60° to obtain a 256 × 192 × 192 volume of data at a spatial resolution of 1 mm^3^.

### 2.5. Imaging Analyses

Analysis methods were performed using FSL 4.1.2 (FMRIB Software Library, Oxford University, Oxford, UK). Prior to multi-subject analyses, each individual functional data set was intensity normalized and registered to high resolution structural and standard space images using 12 parameter optimization methods [54]. Data were smoothed with a Gaussian kernel of 8 mm FWHM and signal from extraneous non-brain tissue in the high-resolution structural images was removed using BET (Brain Extraction Tool) [55]. Statistical analyses were performed using an event-related general linear model approach, as implemented in FEAT (fMRI Expert Analysis Tool) [56]. Group mean statistics for each contrast were generated with a mixed-effects model resulting from the use of within-session variance (*i.e.*, fixed-effects) at the single subject level and between-session variance (*i.e.*, random-effects) at the group level. Statistical parametric maps were computed in FLAME [56]. In the analysis of group mean activation compared to rest for (a) the three rhythm tasks in combination and (b) for each rhythm task and the melody task alone, group statistical parametric maps were thresholded by using clusters determined by a *Z* > 2.3 and a (corrected) cluster significance of *p* < 0.05 [57]. 

## 3. Results

### 3.1. Activity for Tempo, Meter and Pattern Combined

**Figure 2:**
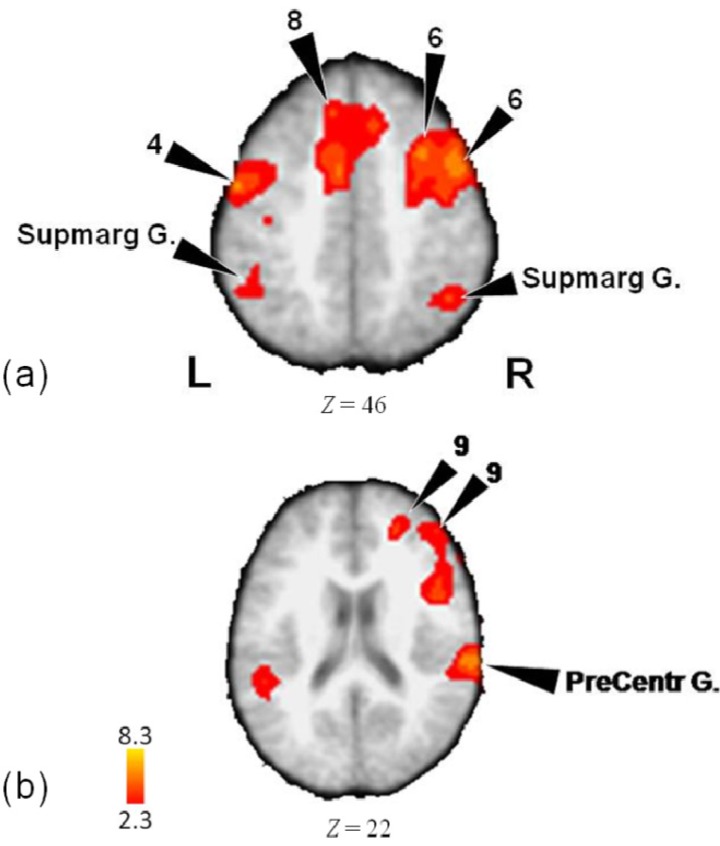
(**a**) Activations in supramarginal and medial frontal gyri, as well as pre-central and middle frontal areas in the analysis of the tempo, meter, and pattern tasks minus rest. The group mean activations are shown in registration with the Montreal Neurological Institute (MNI) 152 standard brain in all figures, with approximate Brodmann areas. Also indicated for each figure is the colour scale of intensity of activations (in Z values). Throughout the figures, the left side of the image is the left side of the brain; (**b**) Activations in inferior frontal, medial frontal, and pre-central gyri in the analysis of the tempo, meter, and pattern tasks minus rest.

The mean activity for all subjects during performance of the three musical rhythm tasks of meter, tempo, and pattern, minus rest, exhibited a distributed set of locations (see Table 1 and Figure 2). There were activations in bilateral supramarginal gyrus (BA 40), right postcentral gyrus (BA 40), right precuneus (BA 7), left superior temporal gyrus (BA 22, 42), right insula (BA 13), predominantly right middle frontal gyrus (BA 6, 8, 10), primarily right medial frontal gyrus (BA 8, 9), right inferior frontal gyrus (BA 45, 9), crus I of right posterior cerebellum, bilateral primary motor cortex (BA 4), and bilateral anterior cingulate (BA 24, 32).

**Table 1:** Stereotactic MNI coordinates, *Z*-score values, and anatomical and Brodmann areas for activations during the Pattern, Meter and Tempo (combined) contrasted to rest.

Lobe	Region	*Z*-Score	*x*	*y*	*z*	BA
*Frontal*						
L	Medial Frontal Gyrus	3.82	0	30	46	8
	Medial Frontal Gyrus	3.67	−8	32	48	8
	Precentral Gyrus	5.23	−56	−8	42	4
	Precentral Gyrus	5.17	−54	−4	48	4
	Precentral Gyrus	3.43	−64	4	42	6
R	Inferior Frontal Gyrus	4.18	48	6	24	9
	Inferior Frontal Gyrus	2.98	46	24	0	45
	Anterior Insula	5.09	46	14	12	13
	Medial Frontal Gyrus	4.22	24	46	22	9
	Medial Frontal Gyrus	3.86	10	26	46	8
	Middle Frontal Gyrus	6.03	48	10	44	6
	Middle Frontal Gyrus	5.59	34	14	52	6
	Middle Frontal Gyrus	3.97	30	−2	54	6
	Middle Frontal Gyrus	3.94	30	−2	50	6
	Middle Frontal Gyrus	3.02	50	28	48	8
	Middle Frontal Gyrus	3.6	46	44	20	10
	Middle Frontal Gyrus	3.5	50	38	24	9
	Precentral Gyrus	4.72	44	−10	46	4
*Limbic*						
L	Anterior Cingulate Gyrus	5.01	−6	2	48	24
R	Anterior Cingulate Gyrus	3.77	10	26	42	32
*Parietal*						
L	Supramarginal Gyrus	4.85	−54	−50	32	40
	Supramarginal Gyrus	4.52	−70	−48	34	40
R	Postcentral Gyrus	5.52	64	−28	20	40
	Precuneus	5.11	24	−64	30	7
	Supramarginal Gyrus	6.65	50	−38	34	40
	Supramarginal Gyrus	5.03	52	−38	38	40
*Temporal*						
L	Superior Temporal Gyrus	5.85	−54	−44	14	22
	Superior Temporal Gyrus	5.71	−78	−24	8	42
	Superior Temporal Gyrus	5.46	−44	−26	6	22
	Superior Temporal Gyrus	5.24	−54	−40	14	22
R	Superior Temporal Gyrus	5.1	50	−20	6	13
*Cerebellum*						
R	Crus I	4.95	42	−64	−34	

### 3.2. Activity for Pattern

**Figure 3:**
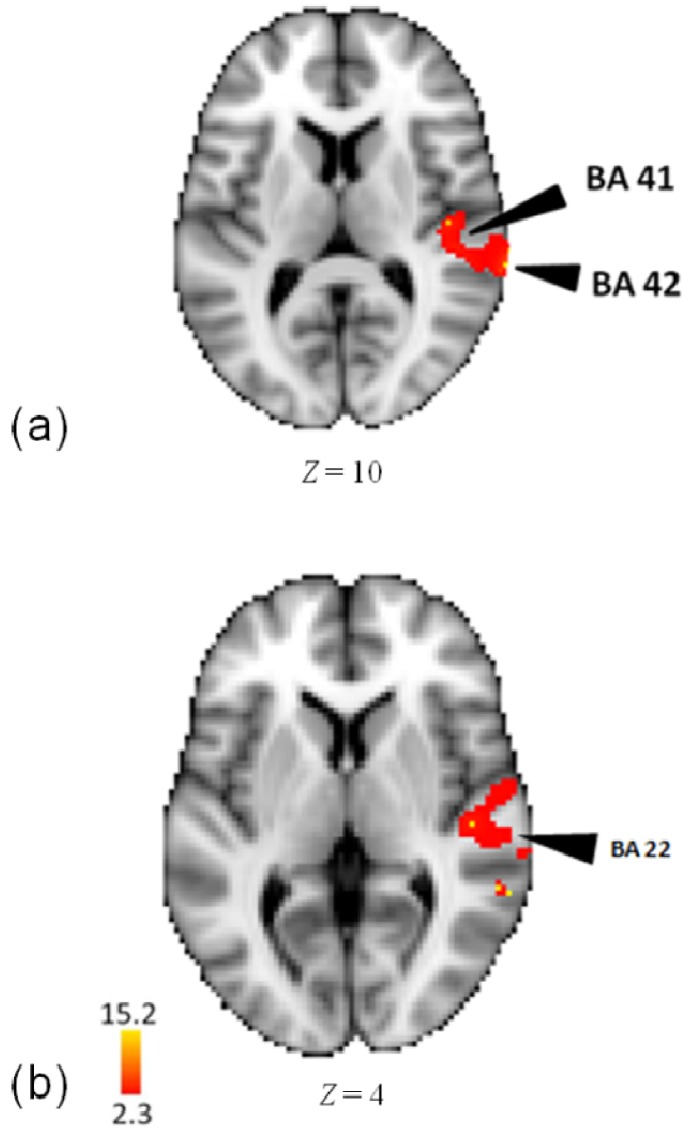
(**a**) Activations in superior and transverse temporal gyri in the analysis of the pattern task minus rest; (**b**) Activations in middle temporal gyrus in the analysis of the pattern task minus rest.

In an analysis of the pattern task, as compared to rest, activity was observed in right areas of middle temporal gyrus (BA 22), superior temporal gyrus (BA 22, 42, 41), and transverse temporal gyrus (BA 42) (see Figure 3 and Table 2).

**Table 2:** Stereotactic MNI coordinates, *Z*-score values, and anatomical and Brodmann areas for activations during the Pattern condition contrasted to rest.

Lobe	Region	*Z*-Score	*x*	*y*	*z*	BA
*Pattern-Rest*						
Temporal	Middle Temporal Gyrus	14.7	62	−46	4	22
R	Middle Temporal Gyrus	14.6	60	−38	6	22
	Superior Temporal Gyrus	15.3	72	−30	10	42
	Superior Temporal Gyrus	14.9	66	−34	6	22
	Superior Temporal Gyrus	14.8	48	−30	14	41
	Transverse Temporal Gyrus	15.1	70	−6	10	42

### 3.3. Activity for Meter

**Figure 4:**
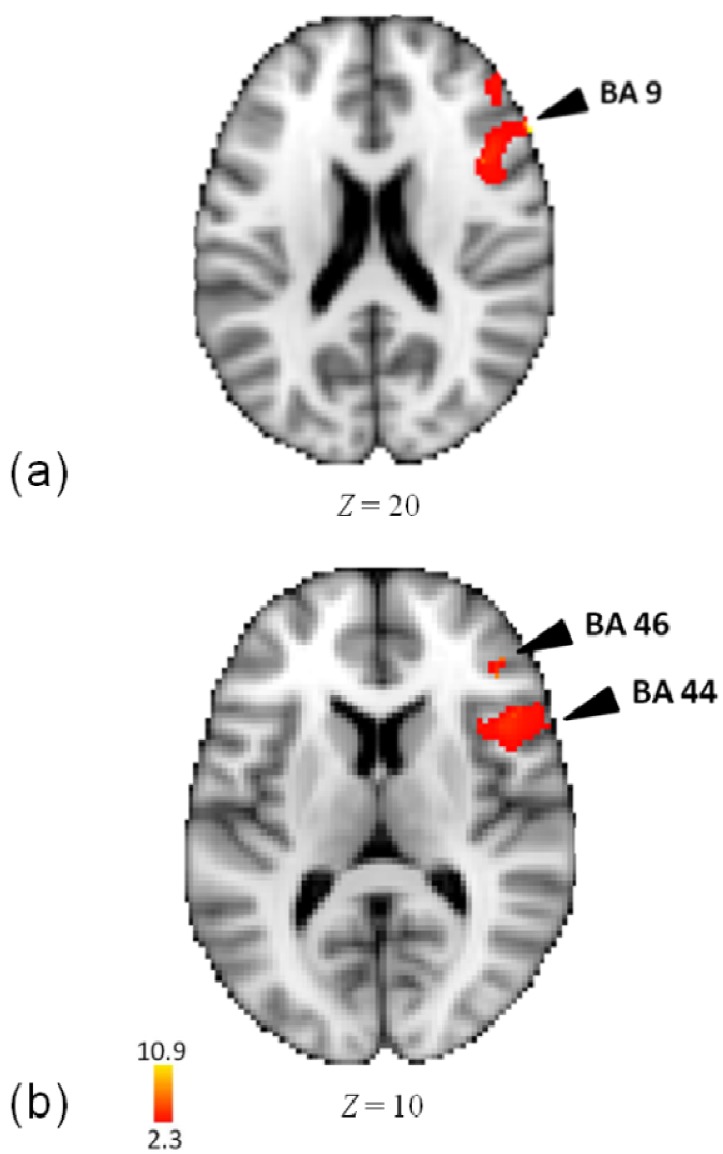
(**a**) Activations in middle and inferior frontal gyri in the analysis of meter task minus rest; (**b**) Activations in inferior frontal gyrus in the analysis of meter task minus rest.

In an analysis of the meter task, as compared to rest, activity was observed in right hemispheric regions of inferior frontal gyrus (BA 44, 9), precentral gyrus (6, 44), and middle frontal gyrus (BA 46) (see Figure 4 and Table 3).

**Table 3:** Stereotactic MNI coordinates, *Z*-score values, and anatomical and Brodmann areas for activations during the Meter condition contrasted to rest.

Lobe	Region	*Z*-Score	*x*	*y*	*z*	BA
*Meter-Rest*						
*Frontal*						
R	Inferior Frontal Gyrus	10.2	60	26	20	9
	Inferior Frontal Gyrus	10.1	60	20	14	44
	Inferior Frontal Gyrus	9.61	56	20	14	44
	Middle Frontal Gyrus	6.72	42	34	10	46
	Precentral Gyrus	11	56	18	4	44
	Precentral Gyrus	6.82	48	4	24	6

### 3.4. Activity for Tempo

**Figure 5:**
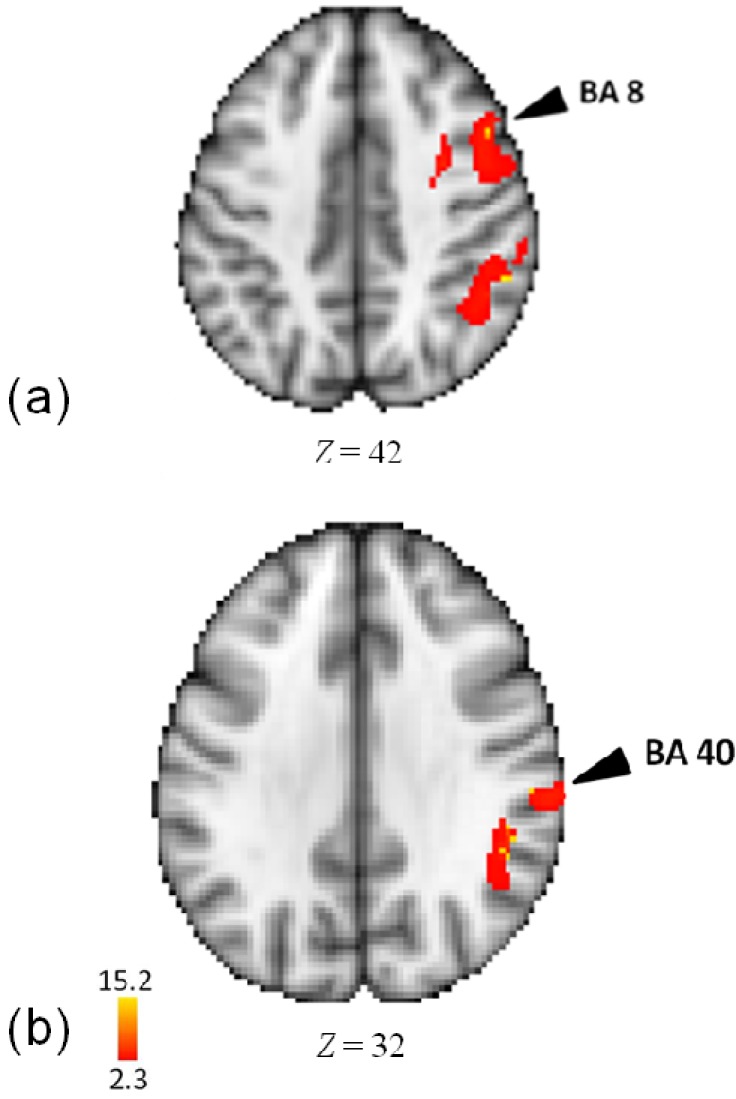
(**a**) Activations in middle frontal gyrus in the analysis of tempo task minus rest; (**b**) Activations in inferior parietal lobule in the analysis of tempo task minus rest.

In an analysis of the tempo task, as compared to rest, activity was observed in right inferior parietal cortex (including supramarginal gyrus), right superior temporal gyrus (BA 22, 42), right insula, and right middle/precentral frontal gyrus (BA 6, 8) (see Figure 5 and Table 4).

**Table 4:** Stereotactic MNI coordinates, *Z*-score values, and anatomical and Brodmann areas for activations during the Tempo condition contrasted to rest.

Lobe	Region	*Z*-Score	*x*	*y*	*z*	BA
*Tempo-Rest*						
*Frontal*						
R	Middle Frontal Gyrus	15.2	30	0	46	6
	Precentral Gyrus	15.2	36	−6	50	6
	Middle Frontal Gyrus	15.1	34	0	54	6
	Middle Frontal Gyrus	14.9	48	12	40	8
	Middle Frontal Gyrus	14.8	34	12	54	6
	Middle Frontal Gyrus	14.7	46	2	46	6
	Posterior Insula	14.6	52	−38	28	13
*Parietal*						
R	Inferior Parietal Lobule	14.9	62	−26	30	40
	Inferior Parietal Lobule	14.8	54	−42	40	40
	Supramarginal Gyrus	14.6	48	−44	34	40
*Temporal*						
R	Superior Temporal Gyrus	15.1	64	−10	2	22
	Superior Temporal Gyrus	14.8	78	−28	20	42

### 3.5. Melody

**Figure 6:**
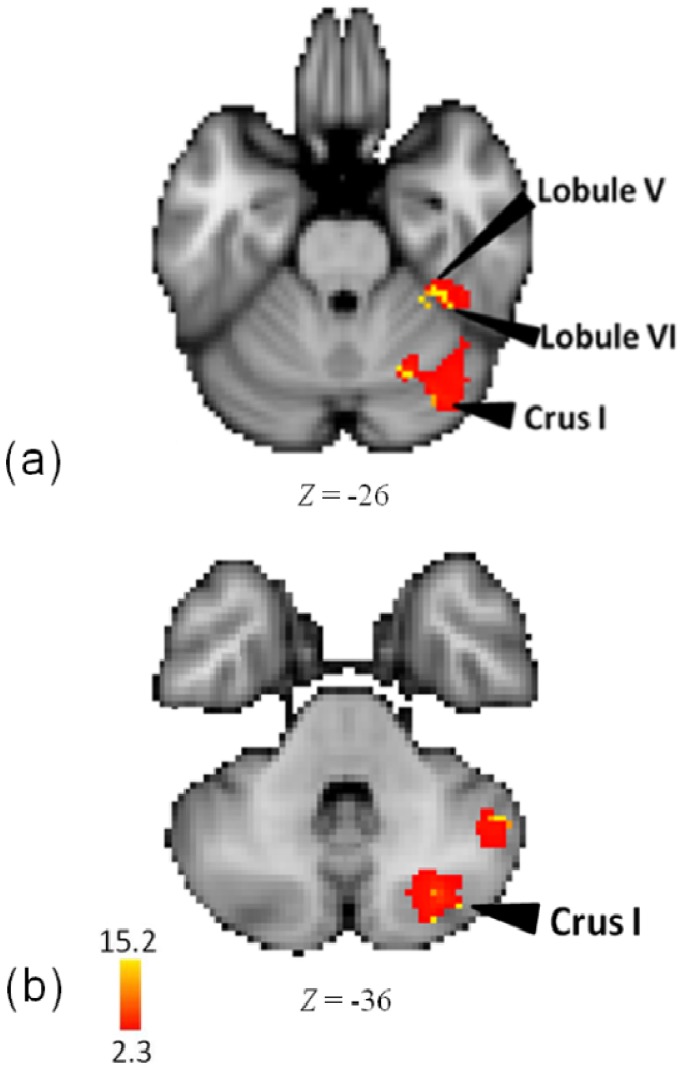
(**a**) Activations in anterior and posterior cerebellum in the analysis of the melody task minus rest; (**b**) Activations in posterior cerebellum in the analysis of the melody task minus rest.

In an analysis of the melody task, as compared to rest, foci were detected in right insula, right postcentral gyrus (BA 40), right claustrum, and right superior temporal gyrus (BA 42). In addition, in right posterior and anterior cerebellum, activity was detected in crus I and lobules VI and V (see Figure 6 and Table 5).

**Table 5:** Stereotactic MNI coordinates, *Z*-score values, and anatomical and Brodmann areas for activations during the Melody condition contrasted to rest.

Lobe	Region	*Z*-Score	*x*	*y*	*z*	BA
*Frontal*						
R	Insula	15.2	46	−14	8	13
	Insula	14.1	42	−12	10	13
*Parietal*						
R	Postcentral Gyrus	14.1	60	−20	20	40
	Postcentral Gyrus	13.9	58	−24	22	40
*Sub-cortical*						
R	Claustrum	14.2	42	−12	6	-
*Temporal*						
R	Superior Temporal Gyrus	15	70	−28	22	42
*Cerebellum*						
R	Lobule V	15.3	34	−38	−26	-
	Lobule VI	14.8	38	−42	−26	-
	Crus I	15.1	36	−78	−36	-
	Lobule VI	14.9	24	−64	−30	-
	Crus I	14.9	36	−74	−28	-
	Crus I	14.8	30	−76	−38	-

### 3.6. Comparing Musicians *vs.* Non-Musicians

We conducted direct group contrasts between musicians and non-musicians, but no clusters reached significance at our pre-determined statistical criteria (cluster-wise correction, see Experimental Section, Imaging Analysis). However, in order to explore trends in those comparisons, we used an uncorrected *Z* value of 2.3, *p* < 0.05. In brief, these trends suggest that musicians seemed to recruit higher-level representations in temporal, occipital, and frontal areas, whereas non-musicians used more sensory-motor, basal ganglia (putamen, caudate), and cerebellar mechanisms. These differences are congruent with comparisons of musicians and non-musicians on pitch memory tasks matched for task difficulty across groups [51]. Further, the pattern of activity in SMA, putamen, posterior cerebellum, and insula in non-musicians suggests the use of a strategy of implicit or sub-vocal counting that was absent in musicians. A reliance on counting by non-musicians, but not musicians, is consistent with the use of elementary strategies rather than more expert, higher cognitive representations. In addition, consistent with earlier studies, non-musicians relied exclusively on right superior and middle temporal cortex, whereas musicians activated predominantly left superior, middle, and inferior temporal cortex.

## 4. Discussion

### 4.1. Common Mechanisms for Pattern, Meter, and Tempo

Common to the processing required for pattern, meter, and tempo in this study are the elements of (a) performing a sensory analysis of the sounds; (b) representing the beat structure (and possibly phrase structure) implicit in those sounds, in either an auditory or motor-sensory (e.g., finger, foot, vocal, or body system or combination thereof) mode; (c) retaining such representations in working memory; (d) comparing features of the first and second sequences on each trial to detect match and mismatches; (e) executive operations for managing ongoing task performance; and (f) potential emotional responses to musical rhythm. These processing mechanisms appear to be reflected in the activities detected for musical rhythm (combined across pattern, meter, and tempo, minus rest) which are mostly distributed in right, or bilateral, areas of frontal, cingulate, parietal, prefrontal, temporal, and posterior cerebellar cortices.

We observed activity in left primary and secondary auditory temporal cortex (BA 42, 22), likely performing early sensory analyses. Comparable activity was observed for passively listening to simple drum patterns (*versus* random rhythm) when combining musicians and non-musicians [24]. There was also activity present in bilateral middle frontal gyrus (BA 8). This is consistent with observations in a PET study of perceptual discrimination of pairs of sequences by non-musicians (−31, 47, 29, as compared to −8, 32, 48) [19]. Thus, this activated area could be involved in representing and controlling the comparison of distinct features of the stimuli. We detected activity in medial, middle, and inferior frontal gyrus (BA 9); no other studies of this kind reported such activity. This area has been linked to working memory [58]. In addition, activity was observed in pre-dominantly right dorsal premotor cortex (primarily in middle frontal gyrus, BA 6). These findings are similar to those in an fMRI study of non-musicians passively listening to musical rhythms (54, −6, 48, as compared to 48, 10, 44 here) [20]. Because this activity is in the right hemisphere of right-handed individuals, it may not relate to implicit motor planning. A more promising possibility relates to evidence that this area is active for updating and representing location in a spatial framework: this mechanism could be used to represent rhythmic features in a spatial format [59].

In addition, there was activity in right lateral frontal pole (BA 10). This is consistent with an fMRI study of pianists playing a musical score of rhythm patterns [60], with a study of non-musicians passively listening to rhythmic auditory patterns [21] and with an fMRI study of non-musicians making button presses to imitate an auditory rhythmic pattern heard a few seconds earlier (45, 51, 13, as compared to 46, 44, 20 here) [61]. This area has been associated with executive function for maintenance of goal and sub-goals, a function that may serve to relate rhythmic phrases and features with the global beat structure in the present tasks [58,62,63]. There was also activity in bilateral primary motor cortex (BA 4), although there were no motor responses in the task. This is consistent with similar activity in passive listening to rhythmic auditory patterns by non-musicians (e.g., −40, 13, 49, as compared to −54, −4, 42 here) [21]. We also detected activity in bilateral anterior cingulate. This area has been linked to error detection in tasks with cognitive load, operations possibly involved in the detection of differences in pairs of rhythmic sequences here [64,65].

There were also activations in parietal cortex in bilateral supramarginal gyrus (BA 40), right postcentral gyrus (BA 40), and right precuneus (BA 7). These are consistent with findings from a case study of a musician with lesions in left inferior parietal lobule who showed impaired discrimination and reproduction of rhythm patterns (while retaining ability to discriminate musical meter) [38]. Our observations in inferior parietal cortex are also similar to those in an fMRI study of non-musicians performing N-back memory tasks with sounds presented at different locations (e.g., 44, −36, 41 and −40, −38, 39, as compared to 52, −38, 38 and −54, −50, 32 here) [66]. These findings may suggest that areas in inferior parietal cortex are involved in representing rhythmic auditory phrases. In addition, the superior parietal lobule has been implicated in working memory operations, which are likely necessary for support of discrimination of pairs of auditory sequences here [67].

In addition, activity was detected in right anterior insula. The insula has been associated with somatosensory representation [68,69,70]. This activity is consistent with other neuroimaging studies [19,71,72]: (1) a study of non-musicians listening to engaging music (32, 13, 2, as compared to 46, 14, 12 here); (2) a study of perceptual discrimination, as here, of pairs of sequences by non-musicians (left insula, −37, 13, −4); and (3) a study of phonemic repetition (−31, 20, 2). Likewise, a study of stroke patients with lesions in insula reported spared auditory function but impaired performance on perception of auditory rhythm and duration [73].

Further, our analyses revealed activity in right crus I in cerebellum. Similar activity emerged in a meta-analysis of many different neuroimaging studies involving a variety of sound processing, common activity was localized in bilateral crus I (*i.e.*, 29, −81, −38, as compared to 42, −64, −34), and to activity localized in right crus I in a meta-analysis of cerebellar activity during functional imaging studies of healthy individuals performing tasks involving working memory (40, −64, −36 as compared to 42, −64, −34 here) [74,75]. Similar evidence confirming a role of the cerebellum in pitch processing is also found in a recent PET study of pitch discrimination in non-musicians [76].

### 4.2. Distinct Mechanisms for Pattern, Meter, and Tempo

Distinctly different brain activity was elicited in each of the conditions for pattern, meter, and tempo processing. In broad outline, brain activity observed here suggests that meter processing recruits a set of mechanisms involved in more cognitive and abstract representations, than does processing pattern or tempo. Pattern processing involves a set of mechanisms involved in auditory information processing, and tempo processing engages mechanisms subserving somatosensory and premotor information. In this sense, meter information in the musical stimulus is initially processed in auditory perception areas of temporal cortex but is then processed more deeply by other sub-systems, whereas pattern information continues to be more deeply processed in different kinds of auditory areas. This is loosely consistent with the view that pattern (phrasing) involves “local” features of the auditory stimulus, whereas tempo, and especially meter, are more abstract (or global) emergent perceptual properties. It is noteworthy that although meter, in contrast to tempo, can seem closely associated with movement, it recruits very few or no motor-related areas, although it engages a variety of apparently somatosensory areas. This possibility is consistent with the observation that in music training and education, meter is often learned by counting, rather than by body feeling and perceived pulse, especially in children.

More specifically, processing musical pattern produced strong activity in right middle and superior temporal cortex (BA 22, 42, 41). On the other hand, processing musical meter elicited strong activity in right prefrontal (BA 9, 46) and right inferior frontal cortex (BA 44, 46). However, processing musical tempo was associated with strong activity in right superior temporal cortex (in regions distinct from those in pattern and meter), right insula, right inferior parietal cortex, supramarginal gyrus (BA 40), and right middle and precentral gyrus (BA 6, 8). In what follows, we examine more closely the functional activities observed here, comparing them to other studies.

#### 4.2.1. Pattern

Processing pattern involves representing temporal intervals at each local time point that vary across segments and must be linked at a higher level of organisation. Our observations suggest that such functions are subserved by selective activity in different regions of right temporal cortex. Specifically, activity in areas of superior and middle temporal cortex may be representing in auditory form the rhythmic phrase structures. This possibility is consistent with fMRI studies of long term memory for music which report activity right superior temporal gyrus at 54, −26, −4, similar to that observed here at 60, −38, 6) [77].

#### 4.2.2. Meter

Meter is an abstract mathematical subdivision of time events like pulses without specified musical content of phrased rhythmic content. Meter as a periodic distribution of isochronous time intervals emerged relatively late in musical culture, and only in Western music (from medieval modal rhythms) [78]. Processing meter involves implicit representation of repeating cycles of the sequential structure of strong and weak beats. This computational activity would seem consistent with the recruitment of prefrontal and frontal areas in right hemisphere. In particular, the areas in prefrontal cortex (BA 9) have been associated with executive control functions involved in operations for branching or nesting sub-goals [58,59,60]. In addition, meter processing activated dorsolateral prefrontal cortex in areas associated with sequential organization (BA 44) and working memory (BA 46).

#### 4.2.3. Tempo

Processing tempo in this study primarily requires representing the change in rate of sounds per unit of time. Such processing may seem consistent with recruitment of mechanisms subserving somatosensory, premotor, and emotion information. In particular, activity in postcentral gyrus and insula are associated with somatosensory or body representation, which may be responsive to changes in tempo (movement to music response) [66,67,68]. Likewise, linking of perceptual and action variables has been linked to areas in inferior parietal cortex near those we observe here (58, −56, 34, as compared to 54, −42, 40 observed here) [79].

The task of monitoring changes in tempo within a sequence likely entailed expectation-driven processing that may recruit mechanisms in lateral middle frontal gyrus (BA 8) (the latter an area sensitive to task uncertainty) [80,81]. Further, monitoring stimulus sequence events has been linked to activity in lateral middle frontal and precentral gyrus (BA 6) [59]. In addition, the processing of tempo activates areas in right superior temporal cortex distinct from those for processing pattern or meter, suggesting a distinct computational process for rate of auditory events. The activity in right superior temporal cortex (BA 22) that we observed is similar to that present in an fMRI study of phonemic repetition in rhythmic patterns (60, −33, 16) [70].

### 4.3. Specific Mechanisms for Melody

Performing our melody task likely involves processing the elements of pitch interval, pitch height, melodic contour, tonal centre, phrase structure, and rhythm, consequently activating emotional and various cognitive mechanisms. These processes seem to be reflected in the activity detected for this task in right parietal, right superior temporal, right insula, right claustrum, and right posterior cerebellum. The pattern of activity for the melody task was generally distinct from those activities for the temporal features. Thus, e.g., the melody task doesn’t much engage prefrontal and frontal areas, as do the temporal structure tasks (compare Table 5 and Table 1). This is generally consistent with earlier findings suggesting dissociations in the neural mechanisms for these two kinds of processing [19,34,35].

Our clusters in crus I (Figure 6) are similar to common activity localized both by a meta-analysis of several neuroimaging studies involving a variety of sound processing [74] (28 −81 −38, as compared to 30 −76 −38 here), and by a meta-analysis of cerebellar activity during functional imaging studies of healthy individuals performing tasks involving emotion processing (26, −64, −34 in [75]). Our cluster in right lobule VI (Figure 6) is similar but homotopic to that in an fMRI study of non-musicians making a same-different discrimination of pairs of melodies (−24, −71, −26 [82], as compared to 24, −64, −30 here). Our responses in right lobule V are very close to common activity localized by a meta−analysis of many different neuroimaging studies involving a variety of sound processing [74] (31, −41, −32, as compared to 34, −38, −26 here). These findings suggest a role for the cerebellum in processing pitch information. The role of the cerebellum in supporting fine auditory pitch perception, like that in our melody task, is confirmed by a study of cerebellar patients with global atrophy who exhibited an impairment in fine pitch perception [10].

The activity in anterior insula also may reflect emotional processing [69], and is similar to those localised in an MEG study of musicians imposing an implicit meter onto isochronous click sequences [83]. The role of the nearby cluster of activity in the claustrum may be to represent auditory events [84] such as the metric beat, and may signal information about that event to other areas through its widespread cortical connectivity.

Our activity in right inferior parietal lobule was near that detected for pattern, tempo, and meter combined (see earlier). The contribution of this parietal activity may be to maintain a spatial representation of the pitch interval and contour information necessary for perceptual discrimination. Our activations in primary auditory cortex (BA 42) were consistent with those in other studies of melody perception [12], and are likely involved in processing pitch information.

### 4.4. General Discussion

One striking feature of these exploratory findings is that pattern, tempo, and meter elicit different distributed neural mechanisms. This suggests a general support circuitry for processing musical rhythm, which is integrated with the areas highlighted in the preceding discussion. These different patterns of activity appear consistent with the different computational requirements of each rhythmic feature. Moreover, these findings refine earlier studies in which features of musical rhythm were combined together [19,20,21]. In addition, our results complement experiments using simpler, less naturally musical rhythms [24,43]. It is notable that meter, in particular, recruited a set of cognitive and executive information processing areas. This seems consistent with the observation that only humans appear to process meter, whereas other species may be equipped to process pattern, and perhaps likewise tempo (a perception of rate *per se*) (*cf.* [17]). From one viewpoint, the (a) simplicity of computation and (b) apparent evolutionary age of mechanisms are confounded in that tempo is a relatively simple computation (*i.e.*, rate) and it activates comparatively old neural systems. By contrast, meter is more complicated and engages both simpler older systems (e.g., basal ganglia) and newer higher cognitive systems (e.g., pre-frontal systems involved in executive function).

During natural music perception, activations representing different features of music occur dynamically within the same time frame. One contribution of the present findings is to suggest how to interpret the function(s) of the components of those activations, particular those outside early core auditory areas. For example, a set of activations may likely be driven jointly by meter and tempo, while another set could likely be driven by pattern alone. The advances suggested here should aid in building system-level models of functional neuroanatomy of rhythm.

### 4.5. Motor-Sensory Representations

Prior research has demonstrated spontaneous activity in motor-related systems when musicians listen to music and when non-musicians passively listening to musical rhythms [20]. In the present study, we had anticipated that pattern processing would likely elicit abstract plans for fine motor tasks (e.g., with voice or fingers) because it is temporally organized at local level, whereas meter and tempo would elicit plans for large scale whole body trunk or foot actions and have a cyclic or repetitive structure. Indeed, we found such differences, for example, when pattern elicited activity in pre-SMA, but meter produced activity in globus pallidus and pre-central gyrus (BA 6). Common to the three rhythm tasks were activations in bilateral motor cortex (BA 4, precentral gyrus) in mouth regions, as well as predominantly right middle frontal cortex (BA 6).

More broadly, the activity pattern common to all three rhythmic conditions, as well as those distinct to each rhythmic feature, indicated a richer set of sensory-related representations (e.g., auditory, motor vocal, motor body, spatial, visual, somotosensory, and emotion) than was present for the melody task. This is consistent with the suggestion that neural representation of temporal features of music are more intrinsic multi-sensory than those of melody and harmony (perhaps tied to melody as a implicit vocal singing, whereas rhythm involves more of the body).

### 4.6. Cerebellum

We observed activity in right posterior cerebellum (crus I) common to meter, pattern, and tempo. There are a variety of hypotheses about cerebellar function in general and about regional specializations within the cerebellum that may bear on interpreting these data [75,85,86]. The functions which could be plausibly involved in performing our tasks would include (a) motor-sensory processing related to mental simulation of action; (b) an involvement in processing the temporal properties of the stimuli; (c) auditory sensory processing; (d) executive functions; (e) processing of perceptual and cognitive information about sequences of events; and (f) emotion processing [10,74,76,87,88,89,90,91,92,93,94,95,96]. The pattern of activity here shows co-activations in crus I coordinated with prefrontal and inferior frontal areas associated with executive functions, including working memory. Recent measures of functional connectivity also suggest a link between prefrontal regions and crus I [86,97,98]. As discussed earlier, there were several different functional areas in cerebellum active during the melody task. Based on a variety of prior findings, these foci can be assimilated to auditory sensory processing, and to a lesser extent to emotion processing.

## 5. Conclusions

The findings in this exploratory study outline some of the distinct neural components of rhythmic structure that may be present in complex interactions when those elements are blended in typical music experiences. Our study is limited by our small sample size of human volunteers and stimulus trials, but has the virtues of being performed in an acoustically quiet setting, unusual for functional neuroimaging studies, and of comparing data within-subject across tasks with different rhythmic features. In addition, as the musicians were exposed to similar but somewhat more complicated stimuli than the non-musicians, the findings for the combination of the musicians and non-musicians likely holds for similarly related materials across a range of varying complexity. The findings here can be evaluated by studies using other techniques in future. In addition, future research could refine these findings via the use of (a) participants with different musical expertise (e.g., drummers *vs.* non-drummer musicians); (b) richer, more natural musical auditory stimuli; (c) musicians from non-Western cultures; (d) tasks providing visual information about the action and instrument used to produce musical sounds; (e) measures of brain activity with higher temporal resolution; and (f) combinations of individual rhythmic features in conjunction with other individual features (melody, timbre, *etc.*). More broadly, these kinds of findings may illustrate how the brain, as reflected in different music cultures, can build different rhythmic architectures by freely combining various components of rhythm as modular building blocks into distinct musical languages.
